# Correction: Carbon nanotube–titanium dioxide nanocomposite support for improved activity and stability of an iridium catalyst toward the oxygen evolution reaction

**DOI:** 10.1039/d2ra90134j

**Published:** 2023-01-12

**Authors:** Eom Ji Kim, Ki hyun Kim, Junu Bak, KwangHo Lee, EunAe Cho

**Affiliations:** a Department of Materials Science and Engineering, Korea Advanced Institute of Science and Technology (KAIST) 291 Daehak-ro, Yuseong-gu Daejeon 34141 Republic of Korea eacho@kaist.ac.kr

## Abstract

Correction for ‘Carbon nanotube–titanium dioxide nanocomposite support for improved activity and stability of an iridium catalyst toward the oxygen evolution reaction’ by Eom Ji Kim *et al.*, *RSC Adv.*, 2022, **12**, 35943–35949, https://doi.org/10.1039/D2RA05027G.

The authors regret the omission of a funding acknowledgement in the original article. This acknowledgement is given below.

This work was partly supported by the National Research Foundation of Korea grant, funded by the Korean Government (MSIT) (NRF2019M3E6A1064710) and by the Korea Institute of Energy Technology Evaluation and Planning (KETEP) grant funded by the Korea government (MOTIE) (20214000000650, Energy Innovation Research Center for Fuel Cell Technology).

The Royal Society of Chemistry apologises for these errors and any consequent inconvenience to authors and readers.

## Supplementary Material

